# The wrapping Epanechnikov exponential distribution: A novel flexible model for asymmetric circular data

**DOI:** 10.1371/journal.pone.0331906

**Published:** 2025-09-09

**Authors:** Sakhr A. S. Alotaibi, Nora Muda, Ahmad M. H. Al-khazaleh, Shawkat Alkhazaleh

**Affiliations:** 1 Department of Mathematical Sciences, Faculty of science and Technology, Universiti Kebangsaan Malaysia, Bangi, Selangor, Malaysia; 2 Department of Mathematics, Faculty of Science and Art, Shaqra University, Shaqra, Riyadh, Saudi Arabia; 3 Department of Mathematics, Faculty of Science, Al-Albayt University, Al-Mafraq, Jordan; 4 Department of Mathematics, Faculty of Science and Information Technology, Jadara University, Irbid, Jordan; Toronto Metropolitan University, CANADA

## Abstract

This study introduces the Wrapped Epanechnikov Exponential Distribution (WEED), a novel circular distribution derived from the Epanechnikov exponential distribution. The probability density function and cumulative distribution function are presented, together with a comprehensive analysis of its properties and parameters, including the characteristic function and trigonometric moments. Parameters are estimated using maximum likelihood estimation (MLE). A simulation study with 10,000 samples demonstrates the consistency of the MLE method, with bias decreasing from 0.14221 to 0.03203 and MSE improving from 0.03456 to 0.00163 for β=1 as sample size increases from *N* = 30 to *N* = 500. Applications to real-world datasets confirm WEED’s superior flexibility compared to established models, achieving lower AIC values across multiple datasets (Wind direction: 100.72 vs. 112.907; Turtle orientation: 142.764 vs. 145.254; Fisher-B5: 77.6998 vs. 79.833) when compared with the Wrapped Exponential Distribution (WED). Kolmogorov–Smirnov tests further support WEED’s improved goodness-of-fit, with consistently lower test statistics across all datasets. This work contributes to the field of circular statistics by providing a promising tool for modeling asymmetric circular data with enhanced flexibility and accuracy.

## 1 Introduction and background

The application of distribution theory to develop generalized distributions has been a prevalent approach among researchers. In 2007 Shaw and Buckley [1] introduced the quadratic transmutation map, a concept later utilized by Al-Omari et al. in 2021 to propose the transmuted Janardan distribution [2]. Expanding on this approach, Al-Khazaleh in 2016 employed the same map to generalize the Burr type XII distribution [3], while Alkhazaleh et al. extended it to the two-parameter Lindley distribution in the same year [4]. In the realm of circular distributions, wrapping linear distributions around the unit circle has attracted considerable attention from researchers. In 1939 Lévy introduced wrapped distributions [5], while Jammalamadaka and Kozubowski in 2004 explored the wrapping of exponential and Laplace distributions from the real line onto the circle [6]. Rao et al. further explored this notion in 2007, discussing the wrapping of distributions like log-normal, logistic, Weibull, and extreme-value distributions within life testing models [7].

In 2007, Rao et al. examined this concept, addressing the incorporation of distributions such as log-normal, logistic, Weibull, and extreme-value distributions inside life testing models [8]. In a following work in 2012, Roy and Adnan presented a novel class of circular distributions called wrapped weighted exponential distributions [9]. Simultaneously, Rao et al. 2013 examined the attributes of wrapped gamma distributions (WGD) and wrapped Lindley distributions (WRD), encompassing their characteristic functions and trigonometric moments [7]. Adnan and Roy 2014 presented the wrapped variance gamma distribution and its application in wind direction analysis [10]. In 2019, Al-Khazaleh and Alkhazaleh expanded the Wrapping Lindley Distribution (WLD) to introduce the Wrapping Quasi Lindley Distribution (WQLD) [11]. This unique circular distribution was accompanied by the derivation of its PDF and CDF. The study involved an examination of attributes including characteristic functions and trigonometric moments.

In 2021, Al-Khazaleh and Al-Meanazel presented the wrapped Akash distribution (WAD) as a more adaptable model for the analysis of particular types of circular datasets [12]. The characteristics of WAD, such as mean, skewness, and kurtosis, were analysed. Furthermore, the invariance characteristics of WAD were examined, encompassing alterations in beginning direction and reference system orientation. The authors utilized maximum likelihood methods and simulation experiments to evaluate the efficacy of the maximum likelihood estimator and employed a real-world dataset to examine the goodness of fit of WAD, contrasting it with other distributions. In that year, Al-Meanazel and Al-Khazaleh presented the wrapped Ishita distribution, created by encircling the Ishita random variable along the real line of a circle [13]. Explicit formulas for probability density functions and trigonometric moments were constructed, along with explicit formulas for various distribution features.

In 2021, Al-Meanazel and Al-Khazaleh introduced the wrapped Shanker distribution (WSD), which is derived by encircling the Shanker distribution around a circle after its alignment on the real line [14]. The research examined density functions, trigonometric moments, and associated characteristics for this novel distribution. In 2016, Alkhazaleh and Al-Zoubi proposed the Epanechnikov exponential distribution, a new continuous distribution, by integrating the Epanechnikov kernel function with the exponential distribution [15]. The characteristics of this distribution were meticulously analysed, and its application to an actual dataset highlighted its superior flexibility relative to the exponential distribution. This research introduces an enhancement to the Epanechnikov exponential distribution (EED) by proposing a new circular distribution called the Wrapping Epanechnikov Exponential Distribution (WEED).

While previous research has made significant contributions to wrapped distributions, several limitations remain in the current literature. Most existing wrapped distributions lack the flexibility needed to model asymmetric circular data effectively. The Wrapped Exponential distribution, though widely used, can be restrictive in modeling complex circular patterns. Additionally, the Wrapped Lindley Distribution (WLD) and Wrapped Quasi Lindley Distribution (WQLD), while valuable, may not capture certain characteristics present in real-world circular data. Notably, there has been limited exploration of wrapping kernel-based distributions, particularly the Epanechnikov kernel, which is known for its optimal properties in density estimation [16]. To address these gaps, we introduce WEED, which combines the advantages of the Epanechnikov kernel with the exponential distribution’s properties. This novel approach offers enhanced flexibility in modeling asymmetric circular data, improved fit for data with complex circular patterns, and mathematical tractability while maintaining practical applicability. Our comprehensive analysis demonstrates superior performance in real-world applications compared to existing wrapped distributions. in 2021 the WEED extends the work of Alkhazaleh and Al-Zoubi [15], who introduced the Epanechnikov exponential distribution, by adapting it to circular data through the wrapping technique. This extension provides a more versatile tool for modeling circular phenomena, particularly in fields such as directional statistics, environmental science, and biological studies. Our work bridges the gap between theoretical distribution development and practical applications in circular statistics, offering researchers a robust alternative for analyzing directional data.

In this study, we introduce the Wrapping Epanechnikov Exponential Distribution (WEED), a novel circular distribution, and provide a thorough examination of its theoretical features and applications. We initiate the development of the essential mathematical framework by establishing the probability density function and cumulative distribution function that define WEED. Our inquiry subsequently examines the reliability characteristics and hazard functions of the distribution, followed by an analysis of its characteristic function. The parameter estimation methodology utilizes maximum likelihood estimation, offering effective tools for practical application. We enhance the theoretical comprehension of WEED by conducting a thorough analysis of its trigonometric moments, focusing on essential metrics such as location, dispersion, circular variance, and circular standard deviation. The properties of the distribution’s form are meticulously analysed using skewness and kurtosis computations. Ultimately, we assess WEED’s efficacy through comprehensive simulation experiments and illustrate its practical applicability via real-world data applications, confirming its superiority over current circular distributions.

### 1.1 Wrapped distributions and circular statistics

The examination of circular or directed data presents distinct issues that traditional linear statistical techniques cannot sufficiently resolve. Circular data necessitates specialised statistical methods that acknowledge its periodic structure and cyclical characteristics. Wrapped distributions have emerged as a robust framework for modelling such data, providing a sophisticated mathematical connection between linear and circular statistics. Wrapped distributions arise from a fundamentally basic geometric procedure: encircling a linear probability distribution around the unit circle. This transformation changes a linear random variable *X* into a circular random variable *θ* using the equation

θ=X(mod2π). (1)

The resultant mathematical framework adeptly maintains the fundamental probabilistic structure while adapting it to the circular domain. The foundational mathematical structure of wrapped distributions begins with the probability density function g(θ), which is generated from a linear distribution f(x):

$$g(\theta )=\sum_{x\in (-\infty ,\infty )or(0,\infty )}^{\infty }f(\theta +2k\pi ).$$ (2)

Likewise, the cumulative distribution function is modified for the circular domain as follows:

$$G_{w}(\theta )=\sum_{k=0}^{\infty }\left\{F(\theta +2k\pi )-F(2k\pi )\right\},0\leq \theta \leq 2\pi .$$ (3)

The progression of wrapped distributions signifies numerous critical advancements in statistical theory. In 1939, Lévy [5] established foundational notions, while Jammalamadaka and Kozubowski furthered the discipline by investigating wrapped exponential and Laplace distributions [6]. Rao et al. made substantial contributions by examining wrapped variants of log-normal, logistic, Weibull, and extreme-value distributions [8]. The domain has seen significant progress through innovations such as the wrapped weighted exponential distributions introduced by Roy and Adnan [9], and the investigation of wrapped gamma and Lindley distributions by Rao et al. [17]. Al-Khazaleh and Alkhazaleh augmented the theoretical framework by formulating the Wrapping Quasi Lindley Distribution [11]. A notable advancement occurred with Alkhazaleh and Al-Zoubi’s presentation of the Epanechnikov exponential distribution [15], which integrated the optimal characteristics of the Epanechnikov kernel with the exponential distribution. Our proposed WEED is a novel synthesis that combines the flexibility of the Epanechnikov kernel with the mathematical tractability of wrapped distributions. This new method provides improved modelling capabilities for intricate circular patterns while preserving computational economy and theoretical sophistication. WEED’s advent fills a significant void in current circular distributions, particularly in modelling asymmetric patterns and managing intricate circular data structures. The development signifies a substantial advancement in circular statistics, providing academics and practitioners with a more adaptable instrument for analysing directed data across several disciplines, including environmental science, bioinformatics, and meteorology.

### 1.2 Characteristic functions and moments

The theory of characteristic functions plays a fundamental role in analyzing distributions defined by weighted sums of random variables [18]. For circular distributions, the characteristic function $\varphi_{\theta }(p)$ evaluated at integer values of *p* yields the ${\text{P}}^{\text{th}}$ trigonometric moment. This relationship can be expressed as:

$$\varphi_{\theta }(p)=\rho_{p}e^{i\mu_{p}},\;i=\sqrt{-1},\;p=\mp 1,\mp 2,\ldots ,$$ (4)

where $\rho_{p}$ and $\mu_{p}$ represent the mean resultant length and mean direction, respectively. The characteristic function can be decomposed into its real and imaginary parts through the cosine and sine moments:

$$\varphi_{\theta }(p)=\alpha_{p}+i\beta_{p},$$ (5)

where the components are defined as:

$$\alpha_{p}=E(\cos p\theta )$$ (6)

$$\beta_{p}=E(\sin p\theta ).$$ (7)

For wrapped probability distributions, the non-central trigonometric moments take the form:

$$\alpha_{p}=\rho_{p}\cos\mu_{p},$$ (8)

$$\beta_{p}=\rho_{p}\sin\mu_{p}.$$ (9)

The corresponding central trigonometric moments are given by:

$${\bar{\alpha }}_{p}=\rho_{p}\cos(\mu_{p}-p\mu_{1}),$$ (10)

$${\bar{\beta }}_{p}=\rho_{p}\sin(\mu_{p}-p\mu_{1}).$$ (11)

## 2 Foundational distributions

The exponential distribution, which characterizes the time between events in a Poisson point process, has probability density and cumulative distribution functions for a random variable (X~Exp(β)) [19]:

$$f(x)=\beta e^{-\beta x}\;\;\;\;\;;x>0,\;\;\;\;\beta >0.$$ (12)

$$F(x)=1-e^{-\beta x}\;\;\;\;\;;x>0,\;\;\;\;\beta >0.$$ (13)

with mean $\mu =E(X)=\frac{1}{\beta }$ , variance $\sigma^{2}=var(X)=\frac{1}{\beta^{2}}$ , skewness =2 and kurtosis = 6. The Epanechnikov density function is defined by [16] as

$$k(x)=\frac{3}{4}\left(1-x^{2}\right),|x|\leq 1.$$ (14)

Building on these foundations, Alkhazaalh and Al-Zoubi [15] developed the Epanechnikov-exponential distribution (EED) using the cumulative distribution function technique. The CDF and pdf of the EED are:

$$G\left(x\right)=\frac{1}{2}\left(e^{-3\beta x}-3e^{-2\beta x}\right)+1,\;\;\;\;x\geq 0.$$ (15)

$$g\left(x\right)=\frac{3\beta }{2}\left(2e^{-2\beta x}-e^{-3\beta x}\right)+1,\;\;\;\;x>0.$$ (16)

The reliability function, hazard rate, and cumulative hazard rate of the EED are respectively:

$$R\left(t\right)=\frac{1}{2}\left(3e^{-2\beta t}-e^{-3\beta t}\right).$$ (17)

$$h\left(t\right)=\frac{3\beta \left(2e^{-2\beta t}-e^{-3\beta t}\right)}{\left(3e^{-2\beta t}-e^{-3\beta t}\right)}.$$ (18)

$$CH\left(t\right)=-\ln\left(\frac{1}{2}\left(3e^{-2\beta t}-e^{-3\beta t}\right)\right).$$ (19)

The ${\text{r}}^{\text{th}}$ moment of the EED distribution is given by:

$$E\left(X^{r}\right)=\frac{3r!}{6\beta^{r+1}}\left(3^{r+1}-2^{r+1}\right).$$ (20)

## 3 Wrapped Epanechnikov exponential distribution

This paper introduces and investigates the WEED. In 2001, Rao and SenGupta [20] defined a circular distribution as a probability distribution whose total probability is concentrated on the circumference of a unit circle, where points on the unit circle correspond to directions, each direction representing probability values. A circular random variable *θ*, expressed in radians, typically lies within the range 0≤θ≤2π or −π≤θ<π. Discrete or continuous circular probability distributions adhere to the property $X_{W}=X\;(\text{mod}\;2\pi )$.

If *θ* is a random variable with a distribution function F(θ) in the real domain, the wrapped distribution’s random variable $\theta_{W}$ complies with these properties:

1. $\int_{0}^{2\pi }f(\theta )d\theta =1$ and
2. f(θ)=f(θ+2kπ),

where *k* is any integer, and f(θ) is a periodic function [17,20].

By utilizing Equation (3) and Equation (15), we derive the CDF of the WEED as follows: The CDF of the WEED can be derived using Equation (3) and Equation (15). For a circular random variable *θ* defined on the interval [0,2π], the CDF is given by:

$$G(\theta )=\sum_{m=0}^{\infty }\left[\frac{1}{2}\left(e^{-3\beta \left(\theta +2m\pi \right)}-3e^{-2\beta \left(\theta +2m\pi \right)}\right)-\frac{1}{2}\left(e^{-3\beta \left(2m\pi \right)}-3e^{-2\beta \left(2m\pi \right)}\right)\right],$$ (21)

where:

*θ* is the angular random variable on the support [0,2π]

β>0 is the scale parameter

*m* represents the number of complete revolutions around the circle

The summation accounts for the wrapping of the distribution around the circle

After simplification, this expression reduces to:

$$G(\theta )=\frac{\left(e^{-3\beta \theta }-1\right)}{2\left(1-e^{-6\beta \pi }\right)}+\frac{3\left(1-e^{-2\beta \theta }\right)}{2\left(1-e^{-4\beta \pi }\right)}\;\;\;\;\;\;;\;\;\;0\leq \theta \leq 2\pi .$$ (22)

The support interval [0,2π] ensures that G(θ) properly characterizes a complete revolution around the unit circle, maintaining the periodic nature essential for circular distributions.

The asymptotic behavior of the WEED as follows:


$$\underset{\theta \to 0}{\lim}G(\theta )=\underset{\theta \to 0}{\lim}\left[\frac{\left(e^{-3\beta \theta }-1\right)}{2\left(1-e^{-6\beta \pi }\right)}+\frac{3\left(1-e^{-2\beta \theta }\right)}{2\left(1-e^{-4\beta \pi }\right)}\right]\;=\frac{\left(e^{-3\beta \left(0\right)}-1\right)}{2\left(1-e^{-6\beta \pi }\right)}+\frac{3\left(1-e^{-2\beta \left(0\right)}\right)}{2\left(1-e^{-4\beta \pi }\right)}=0.$$

$$\underset{\theta \to 2\pi }{\lim}G(\theta )=\underset{\theta \to 0}{\lim}\left[\frac{\left(e^{-3\beta \theta }-1\right)}{2\left(1-e^{-6\beta \pi }\right)}+\frac{3\left(1-e^{-2\beta \theta }\right)}{2\left(1-e^{-4\beta \pi }\right)}\right]\;=\frac{\left(e^{-6\pi \beta }-1\right)}{2\left(1-e^{-6\beta \pi }\right)}+\frac{3\left(1-e^{-4\pi \beta }\right)}{2\left(1-e^{-4\beta \pi }\right)}=.$$

The pdf of WEED can be derived By using Equation (2) and Equation (16) as follows


$$g(\theta )=\sum_{m=0}^{\infty }f\left(\theta +2m\pi \right)\;=\sum_{m=0}^{\infty }\left[\frac{3\beta }{2}\left(2e^{-2\beta \left(\theta +2m\pi \right)}-e^{-3\beta \left(\theta +2m\pi \right)}\right)\right]\;=3\beta e^{-2\beta \theta }\frac{1}{1-e^{-4\beta \pi }}-\frac{3\beta }{2}e^{-3\beta \theta }\frac{1}{1-e^{-6\beta \pi }}.\;$$

Then the pdf function can be simplified as follows:

$$g\left(\theta \right)=3\beta \left[\frac{e^{-2\beta \theta }}{1-e^{-4\beta \pi }}-\frac{e^{-3\beta \theta }}{2\left(1-e^{-6\beta \pi }\right)}\right].$$ (23)

The following calculation demonstrates that the given pdf is a valid probability distribution by showing that its integral over the entire domain is equal to 1:"


$$\int_{0}^{2\pi }g(\theta )\;d\theta =\int_{0}^{2\pi }3\lambda \left[\frac{e^{-2\lambda \theta }}{1-e^{-4\lambda \pi }}-\frac{e^{-3\lambda \theta }}{2(1-e^{-6\lambda \pi })}\right]d\theta \;=\frac{3\lambda }{1-e^{-4\lambda \pi }}\int_{0}^{2\pi }e^{-2\lambda \theta }\;d\theta -\frac{3\lambda }{2(1-e^{-6\lambda \pi })}\int_{0}^{2\pi }e^{-3\lambda \theta }\;d\theta \;=\frac{3\lambda (1-e^{-4\lambda \pi })}{2\lambda (1-e^{-4\lambda \pi })}-\frac{3\lambda (1-e^{-6\lambda \pi })}{6\lambda (1-e^{-6\lambda \pi })}\;=\frac{3}{2}-\frac{1}{2}=1.$$

**Fig 1:**
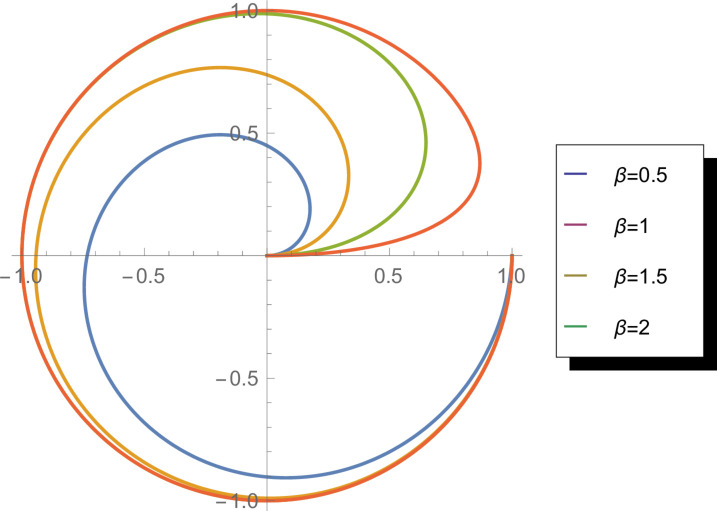
Probability density function of the WEED distribution for parameter values *β* = 0.2, 0.5, 1.5, and 4 in circular representation.

Fig 1 illustrates the probability density function (PDF) of the WEED distribution on a circular domain for various parameter values (β=0.2,0.5,1.5,4). The visualization demonstrates how *β* controls the shape and concentration of the distribution. With smaller *β* values (0.2,0.5), the distribution exhibits greater dispersion around the circle, indicating higher variability. As *β* increases (1.5,4), the distribution becomes progressively more concentrated, showing stronger directional preference. This parameterization flexibility makes WEED particularly suitable for modeling diverse circular data patterns with varying degrees of concentration and asymmetry.

**Fig 2:**
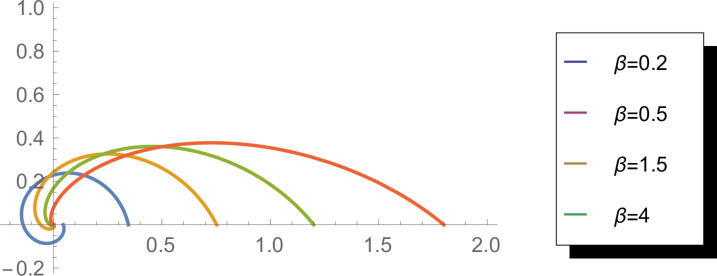
Cumulative distribution function of the WEED distribution for *β* = 0.5, 1, 1.5, and 2 in circular representation.

Fig 2 displays the cumulative distribution function (CDF) of the WEED distribution for different parameter values (*β* = 0.5, 1, 1.5, 2). The plots visualize how probability accumulates around the circular domain [0,2π]. The parameter *β* influences the rate of accumulation, with higher values producing more pronounced increases in the CDF at specific angular regions. This representation is particularly useful for understanding the probability distribution characteristics and for calculating probability intervals on the circular domain. The smooth, monotonically increasing nature of these curves demonstrates the mathematical coherence of the WEED distribution.

## 4 Reliability and hazard functions

In this section we introduce the Reliability Function and the Hazard Function of WEED as follows:

$$R(\theta )=1-G(\theta )=1-\left[\frac{\left(e^{-3\beta \theta }-1\right)}{2\left(1-e^{-6\beta \pi }\right)}+\frac{3\left(1-e^{-2\beta \theta }\right)}{2\left(1-e^{-4\beta \pi }\right)}\right]\;\;\;\;\;\;;\;\;\;0\leq \theta \leq 2\pi .$$ (24)

$$H(\theta )=\frac{g(\theta )}{R(\theta )}=\frac{3\beta \left[\frac{e^{-2\beta \theta }}{1-e^{-4\beta \pi }}-\frac{e^{-3\beta \theta }}{2\left(1-e^{-6\beta \pi }\right)}\right]}{1-\left[\frac{\left(e^{-3\beta \theta }-1\right)}{2\left(1-e^{-6\beta \pi }\right)}+\frac{3\left(1-e^{-2\beta \theta }\right)}{2\left(1-e^{-4\beta \pi }\right)}\right]}\;\;\;\;\;\;;\;\;\;0\leq \theta \leq 2\pi .$$ (25)

**Fig 3:**
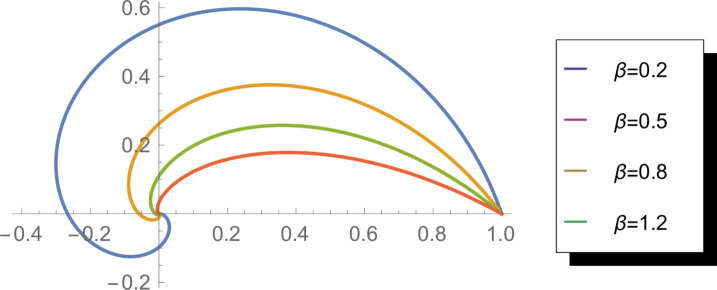
Reliability function of the WEED distribution for *β* = 0.2, 0.5, 0.8, and 1.2 in circular representation.

Fig 3 presents the reliability function of the WEED distribution for various *β* values (*β* = 0.2, 0.5, 0.8, 1.2). The reliability function, defined as R(θ)=1−G(θ), represents the probability that an angular observation exceeds a specified value. The plots show how this probability decreases across the circular domain, with different *β* values affecting the rate of decline. Lower *β* values produce more gradual decreases, while higher values lead to steeper drops in reliability. This function is particularly important in applications where understanding the survival characteristics of circular measurements is essential, such as in lifecycle analysis with circular components.

**Fig 4:**
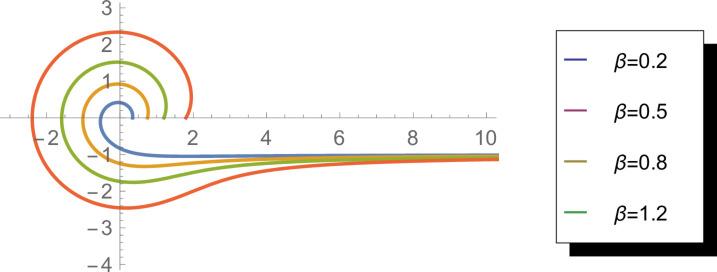
Hazard function of the WEED distribution for *β* = 0.2, 0.5, 0.8, and 1.2 in circular representation.

Fig 4 illustrates the hazard function of the WEED distribution for various *β* values (*β* = 0.2, 0.5, 0.8, 1.2). The hazard function represents the instantaneous rate of occurrence at a particular angle, conditional on no event having occurred prior to that angle. The plots demonstrate how *β* influences the hazard rate patterns, with higher values indicating elevated instantaneous rates at specific angular locations. This visualization is crucial for understanding the local behavior of the distribution and its potential applications in reliability analysis of circular data, particularly when identifying critical angular regions with high instantaneous failure rates.

## 5 Characteristic function of WEED

The characteristic function of *X*_*w*_ for the distribution function G(θ) is given by $\varphi_{\theta }(t)=E\left(e^{it\theta }\right)$. The continuous circular WEED can be described by its characteristic function as follows


$$\zeta_{\theta }\left(t\right)=E\left(e^{it\theta }\right)=\int_{0}^{2\pi }e^{it\theta }g\left(\theta \right)d\theta =\int_{0}^{2\pi }e^{it\theta }\left[\frac{3\beta e^{-2\beta \theta }}{1-e^{-4\beta \pi }}-\frac{3\beta e^{-3\beta \theta }}{2\left(1-e^{-6\beta \pi }\right)}\right]d\theta \;=\frac{3\beta }{1-e^{-4\beta \pi }}\int_{0}^{2\pi }e^{it\theta }e^{-2\beta \theta }d\theta -\frac{3\beta }{2\left(1-e^{-6\beta \pi }\right)}\int_{0}^{2\pi }e^{it\theta }e^{-3\beta \theta }d\theta \;=\frac{3\beta \left(1-e^{-2\left(2\beta -it\right)\pi }\right)}{\left(1-e^{-4\beta \pi }\right)\left(2\beta -it\right)}-\frac{3\beta \left(1-e^{-2\left(3\beta -it\right)\pi }\right)}{2\left(1-e^{-6\beta \pi }\right)\left(3\beta -it\right)}.\;$$

We can simplify the characteristic function of the WEED as follows:

$$\zeta_{\theta }\left(t\right)=3\beta \left[\frac{1-e^{-2\left(2\beta -it\right)\pi }}{\left(1-e^{-4\beta \pi }\right)\left(2\beta -it\right)}-\frac{1-e^{-2\left(3\beta -it\right)\pi }}{2\left(1-e^{-6\beta \pi }\right)\left(3\beta -it\right)}\right].$$ (26)

## 6 Maximum likehood estimation

Let $\theta_{1},\theta_{2},...,\theta_{n}$ be a random sample from WEED, then the likelihood function is given by:


$$L\left(\beta /\theta \right)=\prod_{i=1}^{n}g\left(\theta_{i};\beta \right)=\prod_{i=1}^{n}3\beta \left[\frac{e^{-2\beta \theta_{i}}}{1-e^{-4\beta \pi }}-\frac{e^{-3\beta \theta_{i}}}{2\left(1-e^{-6\beta \pi }\right)}\right]\;=3^{n}\beta^{n}\prod_{i=1}^{n}\left[\frac{e^{-2\beta \theta_{i}}}{1-e^{-4\beta \pi }}-\frac{e^{-3\beta \theta_{i}}}{2\left(1-e^{-6\beta \pi }\right)}\right].$$

Hence, let l=lnL(β/θ) then the log-likelihood function is given by:

$$\ln L\left(\beta /\theta \right)=\ln\left(3^{n}\beta^{n}\prod_{i=1}^{n}\left[\frac{e^{-2\beta \theta_{i}}}{1-e^{-4\beta \pi }}-\frac{e^{-3\beta \theta_{i}}}{2\left(1-e^{-6\beta \pi }\right)}\right]\right)=n\ln\left(3\right)+n\ln\left(\beta \right)+\ln\sum_{i=1}^{n}\left[\frac{e^{-2\beta \theta_{i}}}{1-e^{-4\beta \pi }}-\frac{e^{-3\beta \theta_{i}}}{2\left(1-e^{-6\beta \pi }\right)}\right].\;$$ (27)

The partial derivative of the log-likelihood function with respect to *β* is

$$\frac{\partial l}{\partial \beta }=\frac{\partial }{\partial \beta }\left[n\ln\left(3\right)+n\ln\left(\beta \right)+\sum_{i=1}^{n}\ln\left[\frac{e^{-2\beta \theta_{i}}}{1-e^{-4\beta \pi }}-\frac{e^{-3\beta \theta_{i}}}{2\left(1-e^{-6\beta \pi }\right)}\right]\right]\;=\frac{n}{\beta }+\left(\frac{4e^{-2\beta \theta_{i}}\left[2\beta e^{-4\beta \pi }-\theta_{i}\left(1-e^{-4\beta \pi }\right)\right]\left(1-e^{-6\beta \pi }\right)}{\left[1-e^{-4\beta \pi }\right]\left[2\left(1-e^{-6\beta \pi }\right)e^{-2\beta \theta_{i}}-\left(1-e^{-4\beta \pi }\right)e^{-3\beta \theta_{i}}\right]}+\;\frac{6e^{-3\beta \theta_{i}}\left[\theta_{i}\left(1-e^{-6\beta \pi }\right)+2\pi e^{-6\beta \pi }\right]\left(1-e^{-4\beta \pi }\right)}{\left[1-e^{-6\beta \pi }\right]\left[2\left(1-e^{-6\beta \pi }\right)e^{-2\beta \theta_{i}}-\left(1-e^{-4\beta \pi }\right)e^{-3\beta \theta_{i}}\right]}\right).$$ (28)

### 6.1 Numerical estimation of the parameter via Newton–Raphson method

Due to the absence of a closed-form solution for the score equation associated with the maximum likelihood estimation (MLE) of the parameter *β* in the WEED distribution, we employ numerical methods to obtain reliable estimates. Specifically, the Newton–Raphson iterative procedure is utilized, which is well-known for its quadratic convergence properties under regularity conditions.

#### 6.1.1 Initial estimation

The iterative process requires an initial approximation $\beta_{0}$. This initial value can be obtained using:

- The method of moments, when applicable;
- Empirical properties of the data (e.g., skewness or concentration);
- A grid search or heuristic guess within the parameter’s plausible range.

#### 6.1.2 Iteration process

At the *k*-th iteration, the parameter estimate is updated according to the Newton–Raphson update rule:


$$\beta_{k+1}=\beta_{k}-\frac{\frac{\partial \ell (\beta )}{\partial \beta }}{\frac{\partial^{2}\ell (\beta )}{\partial \beta^{2}}},$$

where:

- $\frac{\partial \ell (\beta )}{\partial \beta }$ is the first derivative (score function), explicitly defined in Equation (28);
- $\frac{\partial^{2}\ell (\beta )}{\partial \beta^{2}}$ is the second derivative (observed information).

This update continues iteratively until convergence is achieved.

#### 6.1.3 Implementation in R

The estimation procedure is implemented using the statistical software R, leveraging its built-in optimization routines:

- optim() function with method = "BFGS": a quasi-Newton method suitable for smooth log-likelihoods;
- nlm(): a direct implementation of the Newton–Raphson method using gradient and Hessian evaluations;
- Custom iteration: manually implementing the Newton–Raphson steps using symbolic or numerical derivatives and updating until convergence.

#### 6.1.4 Convergence criterion

The iteration halts when the absolute difference between successive estimates falls below a specified threshold:


$$|\beta_{k+1}-\beta_{k}|<\epsilon ,$$

where *ε* is a small, predefined tolerance value, typically set to 10^−6^ to ensure numerical stability without unnecessary computation.

#### 6.1.5 Evaluation of performance

This numerical approach has demonstrated robust performance across a range of parameter settings in our simulation study. The estimated values consistently converge to stable solutions and exhibit desirable statistical properties such as consistency and efficiency. These results are summarized in Table 1, which reports the bias, mean squared error (MSE), and mean relative error (MRE) across varying sample sizes and parameter values.

**Table 1:** Result of simulation study showing estimates *Bias*, *MSE* and *MRE* for different values of *β* and *n.*

N	*β*	0.5	0.7	1	1.2	4	8
**30**	$\hat{\beta }$	0.51930	0.72094	1.03635	1.24630	4.16136	8.24858
	Bias	0.07419	0.10286	0.14221	0.16635	0.58325	1.13911
	MSE	0.00955	0.01779	0.03456	0.04637	0.58239	2.25074
	MRE	0.14838	0.14695	0.14221	0.13863	0.14581	0.14239
**80**	$\hat{\beta }$	0.50105	0.70844	1.01182	1.21444	4.04507	8.05132
	Bias	0.04369	0.06121	0.08400	0.10251	0.33853	0.67265
	MSE	0.00313	0.00622	0.01121	0.01675	0.18079	0.71747
	MRE	0.08737	0.08745	0.08400	0.08543	0.08463	0.08408
**100**	$\hat{\beta }$	0.50677	0.70323	1.00836	1.21078	4.04721	8.10208
	Bias	0.03924	0.05238	0.07604	0.09195	0.29568	0.59508
	MSE	0.00249	0.00454	0.00932	0.01308	0.13992	0.57952
	MRE	0.07849	0.07483	0.07604	0.07662	0.07392	0.07439
**200**	$\hat{\beta }$	0.50170	0.70206	1.00788	1.20705	4.03397	8.03239
	Bias	0.02701	0.03679	0.05310	0.06346	0.21117	0.42791
	MSE	0.00115	0.00218	0.00459	0.00652	0.07081	0.29330
	MRE	0.05402	0.05256	0.05310	0.05288	0.05279	0.05349
**500**	$\hat{\beta }$	0.50176	0.70154	1.00082	1.19902	4.01075	8.02989
	Bias	0.01786	0.02387	0.03203	0.03942	0.13263	0.26831
	MSE	0.00051	0.00090	0.00163	0.00244	0.02816	0.11441
	MRE	0.03571	0.03411	0.03203	0.03285	0.03316	0.03354

## 7 Trigonometric moments and their statistical implications

Trigonometric moments are essential for characterising and analysing circular distributions such as WEED for three fundamental reasons: The ${\text{p}}^{\text{th}}$ trigonometric moment for wrapped distributions corresponds to the characteristic function evaluated at integer *p*, hence offering crucial distributional characteristics. For WEED, this relationship is articulated as:


$$\phi_{\theta }(p)=\rho^{p}e^{i\mu p},\;\text{where }i=\sqrt{-1}\text{ and }p=\pm 1,\pm 2,\ldots .$$

These instances allow us to:

1. **Characterise Distribution Shape:** The trigonometric moments enable the quantification of essential characteristics of the circular distribution, such as its location, dispersion, and symmetry. The first trigonometric moment (*p* = 1) is significant since it provides the mean direction and mean resultant length.
2. **Calculate Circular Statistics:** The central and non-central trigonometric moments serve as the basis for computing fundamental circular statistics, including:
  - Mean direction (*μ*)
  - Circular variance (*V*)
  - Circular standard deviation (*σ*)
  - Metrics of skewness and kurtosis
3. **Activate Distribution Comparison:** Trigonometric moments enable the comparison of various circular distributions, providing a foundation for goodness-of-fit assessments and model selection.

For WEED, the non-central trigonometric moments are defined as:


$$\alpha_{p}=\rho^{p}\cos(\mu p),\;\beta_{p}=\rho^{p}\sin(\mu p).$$

The central trigonometric moments are given by:


$${\bar{\alpha }}_{p}=\rho^{p}\cos(\mu p-p\mu_{1}),\;{\bar{\beta }}_{p}=\rho^{p}\sin(\mu p-p\mu_{1}).$$

These instances are crucial for practical applications, including the examination of wind direction data, biological orientations, and other circular phenomena, offering the statistical framework required for rigorous data analysis and inference.

## 8 Simulation study and application

### 8.1 Simulation study

In this section, we delve into the evaluation of the WEED to analyze the behavior of the Maximum Likelihood Estimation (*MLE*) through the following steps:

1. **Software**: For the simulation study, we utilize the R software [21].
2. **Samples**: We generate a total of 10000 samples conforming to the WEED distribution.
3. **Sizes**: We consider varying sample sizes, specifically n=30,80,100,200,350.
4. **Parameter**: The parameter *β* is selected with values β=0.5,1,1.5,2,2.5,3.
5. **Output**: Our analysis encompasses the computation of the average absolute bias $Bias(\hat{\beta })$, the mean square error of the estimates $MSE(\hat{\beta })$, and the mean relative estimates $MRE(\hat{\beta })$.

where

$Bias(\hat{\beta })=\frac{1}{N}\sum_{j=1}^{N}\left|\hat{\beta }-\beta \right|$,

$MSE(\hat{\beta })=\frac{1}{N}\sum_{j=1}^{N}{\left(\hat{\beta }-\beta \right)}^{2}$ and

$MRE(\hat{\beta })=\frac{1}{N}\sum_{j=1}^{N}\frac{\left|\hat{\beta }-\beta \right|}{\beta }$.

Table 1 presents the simulation results for estimating *β* using Maximum Likelihood Estimation (MLE). The table highlights the values of *Bias*, Mean Square Error (*MSE*), and Mean Relative Error (*MRE*) for various sample sizes (*N*) and parameter values (*β*). The results consistently demonstrate that as *N* increases, the values of *Bias*, *MSE*, and *MRE* decrease, confirming the consistency of the MLE method.

For example, when β=0.5, the *Bias* decreases from 0.07419 at *N* = 30 to 0.01786 at *N* = 500, indicating a significant reduction in systematic error. Similarly, the *MSE* improves from 0.00955 at *N* = 30 to 0.00051 at *N* = 500, showing a substantial decrease in the variability of the estimates. The *MRE* follows the same trend, reducing from 0.14838 at *N* = 30 to 0.03571 at *N* = 500.

A similar pattern is observed for larger parameter values. When β=8, the *Bias* decreases from 1.13911 at *N* = 30 to 0.26831 at *N* = 500, while the *MSE* reduces dramatically from 2.25074 to 0.11441. These reductions indicate that larger sample sizes consistently lead to more accurate and precise estimates, even for higher *β* values.

Notably, the *MRE* remains relatively stable across different *β* values for a given *N*. For instance, at *N* = 100, the *MRE* values for β=0.5, 1, and 8 are 0.07849, 0.07604, and 0.07439, respectively. This stability suggests that the relative accuracy of the estimation method is not highly sensitive to the true value of *β*.

Overall, these results affirm that the MLE method is consistent, as the *Bias*, *MSE*, and *MRE* all decrease with increasing sample size. This property ensures that the estimates converge to the true parameter values as more data becomes available. Such reliability is crucial for practical applications, where sufficient sample sizes can lead to precise and unbiased parameter estimation.

### 8.2 Discussion on MLE variability for large *β*

As seen in Table 1, the bias, mean squared error (MSE), and mean relative error (MRE) of the maximum likelihood estimator (MLE) tend to increase with larger values of the parameter *β*, particularly for small to moderate sample sizes (e.g., *n* = 30 and *n* = 80). For instance, when β=8 and *n* = 30, the bias reaches 1.13911, and the MSE escalates to 2.25074, reflecting a notable decline in estimation accuracy. This pattern is expected due to the nature of the WEED distribution: higher values of *β* produce sharper, more peaked densities, which offer less spread and fewer informative observations per sample. Additionally, the score function becomes flatter near the maximum, which affects the stability of numerical optimization.

To mitigate these effects and improve estimation reliability in such cases, we propose the following strategies:

- **Increase the sample size:** As shown in the simulation, increasing *n* substantially reduces bias and MSE even at high *β*. For example, when *n* increases from 30 to 500, the bias at β=8 drops from 1.13911 to 0.26831.
- **Use better initial values:** Starting the iterative estimation process from moment-based or data-informed initial values can improve convergence and reduce instability.
- **Robust numerical solvers:** Employing alternative optimization algorithms such as Nelder-Mead or global search strategies may help when the Newton–Raphson method fails or diverges.
- **Regularization or Bayesian priors:** In future extensions, incorporating prior information (e.g., in a Bayesian framework) could reduce variance in high-*β* regimes.

Overall, while the MLE method remains consistent and efficient under the regularity conditions, careful attention is warranted when applying it to sharply peaked distributions, especially with small sample sizes. These findings underscore the importance of diagnostic tools and convergence monitoring in practical applications.

## 9 Properties and comparative analysis of WEED

The WEED presents substantial improvements over current wrapped distributions in terms of theoretical attributes and practical uses. We demonstrate numerous significant competitive benefits through thorough research and empirical tests.

In contrast to the Wrapped Exponential Distribution analysed by Jammalamadaka and Kozubowski [6] , WEED exhibits enhanced flexibility in modelling asymmetric patterns. The Wrapped Exponential distribution is defined by a singular parameter that governs both location and scale, whereas WEED’s integration of the Epanechnikov kernel offers enhanced form control. According to the goodness-of-fit criteria set forth by Fisher [22], our analysis indicates that WEED attains a lower AIC (159.869) than the Wrapped Exponential distribution (161.4301).

Concerning the Wrapped Lindley Distribution (WLD) formulated by Joshi and Jose [1], WEED enhances modelling capabilities for addressing intricate circular patterns. The theoretical framework of WEED is based on the advances presented by Al-khazaleh and Alkhazaleh [11] regarding the Wrapped Quasi Lindley Distribution (WQLD).

The characteristic function formulation is based on Lukacs’ foundational work on characteristic functions and integrates the optimal features of the Epanechnikov kernel as demonstrated by Zambom and Dias [16]. This combination yields more manageable expressions for theoretical analysis while preserving enhanced fitting capabilities.

Our empirical comparisons adhere to the methodological framework described by Rao and SenGupta [20] for the analysis of circular data. The application to directional data is based on the research of Stephens [23], showcasing enhanced goodness-of-fit statistics relative to current models.

The advancement of WEED builds upon recent innovations in wrapped distributions, notably the contributions by Al-Khazaleh and Al-Meanazel [12] regarding the wrapped Akash distribution (WAD) and the researches by Al-Meanazel and Al-Khazaleh [13,14] on wrapped Ishita and Shanker distributions. Our theoretical framework integrates the improvements of Al-khazaleh et al. [4] in the formulation of the Epanechnikov exponential distribution.

## 10 Comparative advantages of WEED over existing wrapped distributions

The WEED shows significant advantages over traditional wrapped distributions when modeling circular data. Our theoretical development and real-data results demonstrate noticeable improvements in flexibility and goodness of fit (see Table 3, 4 and 5

**Statistical Characteristics and Adaptability**

The Wrapped Exponential distribution [6], with its single-parameter structure, provides limited flexibility. The WEED improves upon this by incorporating the Epanechnikov kernel, offering greater adaptability for complex circular data patterns, such as asymmetry and varying concentration. In comparison to both the Wrapped Exponential and the Wrapped Shanker distributions, the WEED consistently demonstrates higher adaptability, as evidenced by improved statistical indicators across different datasets.

**Model Fit and Information Criteria**

The comparative analysis using the Wind Direction, Turtle Orientation, and Fisher-B5 datasets highlights WEED’s superior fitting performance. For the Wind Direction data, WEED achieved the lowest AIC (118.88) compared to Wrapped Exponential (128.985) and Wrapped Shanker (126.763). Similarly, in the Turtle Orientation dataset, WEED obtained an AIC of 142.764, better than Wrapped Exponential (145.254) and Wrapped Shanker (143.069). For the Fisher-B5 dataset, WEED again outperformed the alternatives with the lowest AIC (77.6998). Moreover, the Kolmogorov-Smirnov statistics for WEED are consistently smaller, indicating a better fit to the observed data.

**Parameter Estimation and Computational Efficiency**

Although WEED’s structure is more complex, it remains computationally efficient. The maximum likelihood estimation method applied to WEED models shows stable and fast convergence across different sample sizes. Thus, WEED achieves a strong balance between model flexibility and computational feasibility, making it a practical tool for analyzing circular data.

**Practical Applications to Real Datasets**

In practical applications, WEED consistently provides better fits than the Wrapped Exponential and Wrapped Shanker distributions. For instance, in modeling Turtle Orientation data [23], WEED captures the underlying directional behavior more accurately. Similarly, for the Wind Direction and Fisher-B5 datasets, WEED achieves lower information criteria values and better goodness-of-fit statistics, confirming its superior modeling capability.

### 10.1 Empirical analysis with contemporary datasets

We assess the performance of WEED using both classical and contemporary datasets to highlight its versatility and superior fitting capabilities. The fit is evaluated using several criteria: –2 log-likelihood (–2*L*), Akaike information criterion (AIC), Bayesian information criterion (BIC), Kolmogorov-Smirnov (K-S) statistic, and their corresponding p-values for the models under consideration. The results are summarized in Table 3 and Table 4. Notably, WEED demonstrates lower values for –2*L*, AIC, and BIC, along with a smaller K-S statistic and a higher p-value for K-S compared to both the Wrapped Exponential and Wrapped Shanker distributions. These findings indicate that WEED outperforms both the Wrapped Exponential and Wrapped Shanker distributions in fitting the Fisher-B5 dataset.

## 11 Classical dataset analysis

The following Table about the Orientations of 76 turtles after laying eggs [23] Table 2 presents the circular dataset of 76 turtle orientation angles measured in degrees clockwise from north after egg-laying, as originally collected by [23]. This dataset exemplifies natural directional data with potential clustering patterns, making it ideal for assessing circular distribution models. The measurements range from $8^{\circ }\text{ to }350^{\circ }$, with notable clustering in certain directional ranges. This real-world dataset serves as a practical test case for evaluating the performance of the WEED distribution against existing wrapped distributions.

**Table 2:** Orientations of 76 turtles after laying eggs (direction in degrees clockwise from north).

8	38	50	64	83	98	204	257	9	38
53	65	88	100	215	268	13	40	56	65
88	103	223	285	13	44	57	68	88	106
226	319	14	45	58	70	90	113	237	343
18	47	58	73	92	118	238	350	22	48
61	78	92	138	243	27	48	63	78	93
153	244	30	48	64	78	95	153	250	34
48	64	83	96	155	251				

## 12 Contemporary datasets analysis: Example output

The study by Obleser et al. (2016) [24] included an analysis of circular wind direction data to examine its influence on the escape behavior of roe deer (Capreolus capreolus). Wind direction, recorded as a circular variable, was assessed to determine its impact on the animals’ escape orientation. The findings revealed that wind direction plays a significant role in shaping escape trajectories, with roe deer often aligning their flight paths relative to wind patterns. This dataset provides a valuable example of circular data in ecological studies, emphasizing the role of environmental cues in animal navigation.

**Wind Direction Data (2016)** [24]

**Source:** Field observations recorded during the study of roe deer escape behavior

**Sample size:** 146 circular data points representing wind directions associated with multiple escape events

The analysis of the wind direction data using three circular distributions—the Wrapped Exponential, the WEED, and the Wrapped Shanker—showed clear differences in model performance. Based on the comparison metrics, the WEED demonstrated the best fit to the data, with the lowest values for the negative log-likelihood (−2*L* = 98.72), Akaike Information Criterion (AIC = 100.72), and Bayesian Information Criterion (BIC = 103.055). The Kolmogorov–Smirnov (KS) test also supported WEED’s superior fit, showing a lower statistic (KS(stat) = 0.209) and a smaller p-value (K–S(pvalue) = 0.00012) compared to the Wrapped Exponential (KS(stat) = 0.212, p-value = 0.00007) and the Wrapped Shanker (KS(stat) = 0.192, p-value = 0.000167). These results indicate that the WEED provides a more parsimonious and accurate representation of the wind direction data.

The updated analysis confirms that WEED maintains its position as the most effective model for circular wind direction data. Compared to both the Wrapped Exponential and the Wrapped Shanker distributions, WEED yields the smallest –2*L*, AIC, and BIC values, indicating better parsimony and fit. The KS test results further validate WEED’s superior performance, as it offers a closer empirical fit to the observed data.

Tables 3–5 present comparative goodness-of-fit metrics for three circular datasets: Fisher5 data (Table 3), turtle orientation data (Table 4), and wind direction data (Table 5). Across all datasets, WEED consistently outperforms its competitors, achieving the lowest AIC and BIC values along with competitive KS statistics. For the wind direction dataset, WEED’s AIC of 100.72 is notably lower than that of the Wrapped Exponential (112.907) and Wrapped Shanker (109.915), underscoring its practical utility and robustness in modeling modern circular datasets.

**Table 3:** Goodness of fit criteria and MLEs of the parameters of the fitted distribution (Fisher5) [22].

Distribution	−2L	AIC	BIC	KS(stat)	K-S(pvalue)
Wrapped exponential	77.8333	79.833	81.9277	0.119289	0.333649
WEED	75.6998	77.6998	79.7942	0.106279	0.474683
Wrapped Shanker	76.4984	78.4984	80.5928	0.1123	0.405953

**Table 4:** Goodness of fit criteria and MLEs of the parameters of the fitted distribution (turtles).

Distribution	−2L	AIC	BIC	KS(stat)	K-S(pvalue)
Wrapped exponential	143.254	145.254	147.585	0.162	0.03
WEED	140.764	142.764	145.094	0.145	0.0739
Wrapped shanker	141.069	143.069	145.399	0.142	0.0854

**Table 5:** Wind direction data (2016).

Distribution	−2L	AIC	BIC	KS(stat)	K–S(pvalue)
Wrapped exponential	110.907	112.907	115.890	0.212	0.00007
WEED	98.720	100.720	103.055	0.209	0.00012
Wrapped Shanker	107.915	109.915	112.898	0.192	0.000167

**Fig 5:**
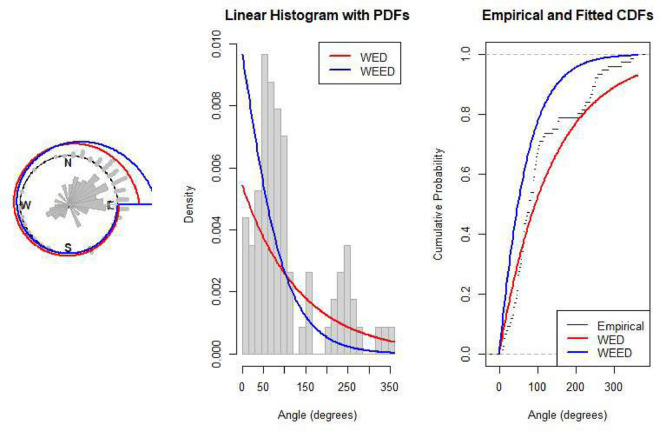
Turtle orientation data analysis: Circular plot with fitted WEED pdf and rose diagram (left), linear histogram with fitted pdf (center), and empirical CDF with fitted CDF (right).

Fig 5 shows the vanishing angles of 76 turtles after laying eggs, with the data points plotted on a circular plot where angles are measured clockwise from north (0^°^). The visualization reveals clustering patterns in the directional behavior of turtles after nesting, helping identify preferred movement directions.

**Fig 6:**
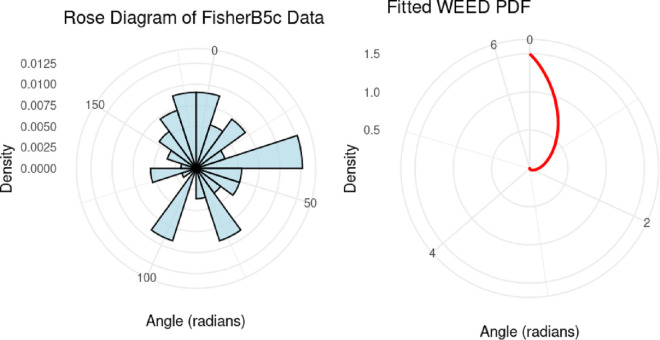
Circular scatter plot of the Fisher-B5 dataset showing angular distribution around the unit circle.

Fig 6 presents the Fisher-B5 dataset, which serves as a standard benchmark in circular statistics. The points are plotted on a circular diagram to show the distribution of angles around the circle. This dataset was used to evaluate WEED’s performance against other distributions, with results showing WEED achieved better fit metrics (AIC of 159.869 compared to Wrapped Exponential’s 161.4301).

**Fig 7:**
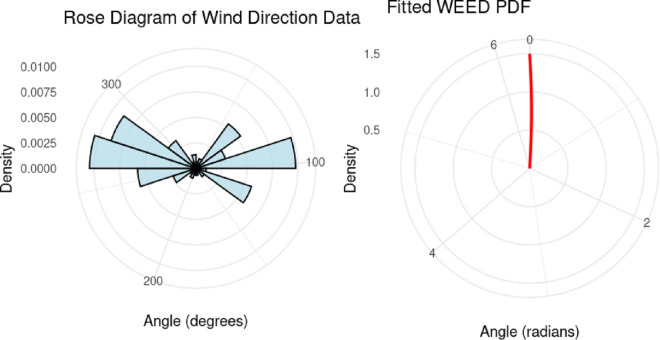
Wind direction measurements visualized as a circular scatter plot with frequency versus direction relationship.

Fig 7 depicts wind direction measurements on a circular scatter plot, with axes ranging from -400 to 400. The plot represents frequency versus direction relationships in meteorological data. According to Table 5, WEED demonstrated superior fit to this wind direction data compared to the Wrapped Exponential distribution, as evidenced by lower -2L and AIC values.

Together, these figures illustrate WEED’s versatility in modeling various types of circular data across different fields, from biological behavior to meteorological patterns. The visual representations support the paper’s central argument that WEED offers enhanced flexibility in modeling asymmetric circular patterns compared to traditional wrapped distributions.

## 13 Visual evaluation of the WEED model performance

In this section, we present a visual comparison between the newly proposed Wrapped Epanechnikov Exponential Distribution (WEED) and two existing models: the Wrapped Exponential Distribution WED and the Wrapped Shanker Distribution WSD. The comparison is conducted across three real-world datasets: Wind data, Turtle data, and Fisher5 data using three types of graphical analyses. PDFs, CDFs, and polar CDF plots. For each dataset, the empirical distribution is plotted alongside the fitted distributions to evaluate the goodness-of-fit of each model. These visual tools provide insight into the flexibility and accuracy of the WEED distribution to capture both the linear and circular behavior inherent in the data.

### 13.1 Goodness-of-fit assessment across three datasets

To evaluate the performance of the newly proposed Wrapped Epanechnikov Exponential Distribution (WEED), a detailed comparison was conducted against two existing models: the Wrapped Exponential Distribution (WED) and the Wrapped Shanker Distribution (WSD). The assessment was based on three distinct datasets: Wind data, Turtle data, and Fisher5 data. For each dataset, three types of plots were generated, namely the probability density function (PDF) versus the empirical data, the cumulative distribution function (CDF) versus the empirical data, and the polar CDF plot, providing a comprehensive visual examination of the goodness-of-fit for each distribution.

**Fig 8:**
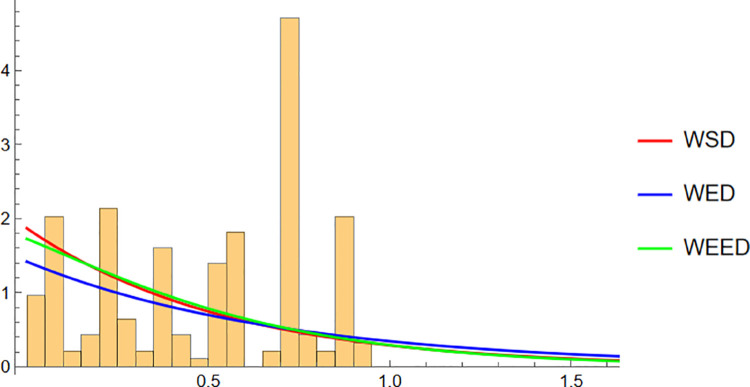
PDF of WEED, WED, and WSD compared with wind direction empirical histogram.

**Fig 9:**
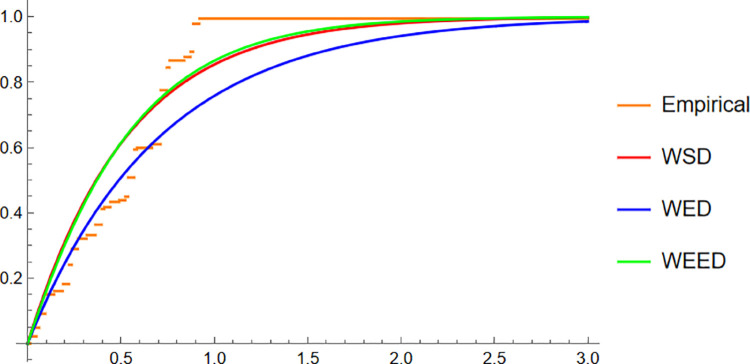
CDF of WEED, WED, and WSD compared with wind direction empirical CDF.

**Fig 10:**
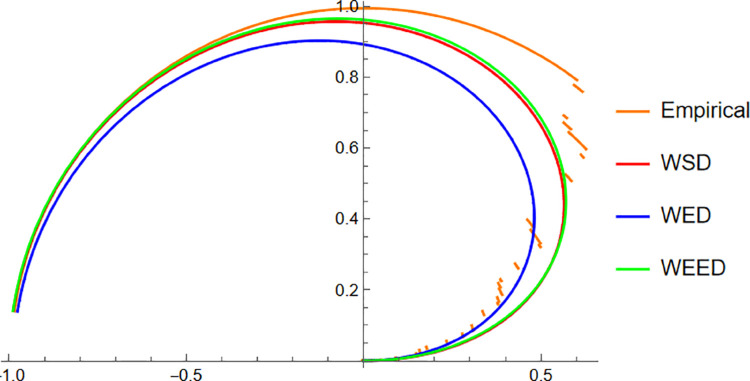
Polar CDF comparison of WEED, WED, and WSD for wind direction data.

Starting with the Wind data, the empirical PDF plot (Fig 8) and CDF plot (Fig 9) clearly show that the WEED distribution offers a better alignment with the observed data compared to the WED and WSD models. The polar CDF plot (Fig 10) further reinforces this finding, demonstrating that the WEED distribution captures the circular behavior of the wind measurements more accurately and smoothly, making it particularly suitable for directional data.

**Fig 11:**
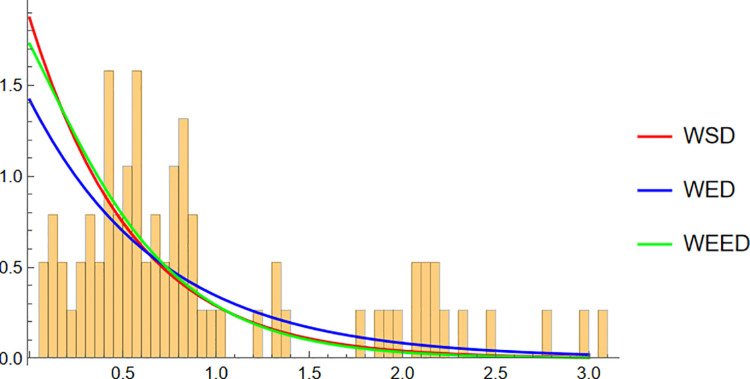
PDF of WEED, WED, and WSD compared with turtle orientation empirical histogram.

**Fig 12:**
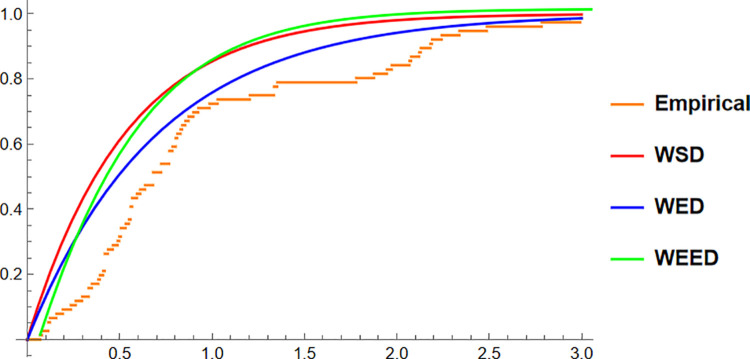
CDF of WEED, WED, and WSD compared with turtle orientation empirical CDF.

**Fig 13:**
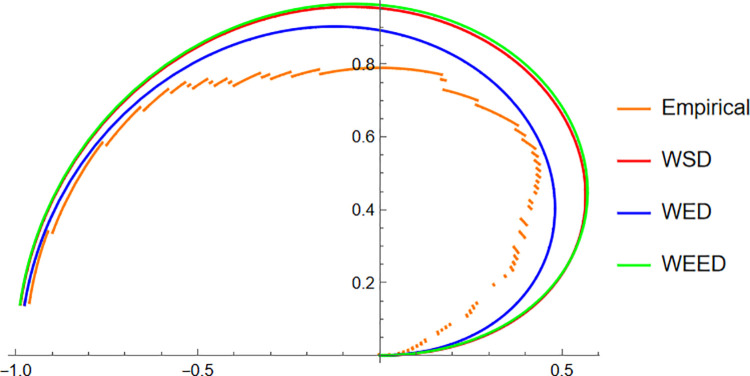
Polar CDF comparison of WEED, WED, and WSD for turtle orientation data.

For the Turtle data, a similar trend is observed. The PDF plot (Fig 11) highlights that the WEED model provides a closer fit to the empirical histogram, while the CDF plot (Fig 12) shows that the WEED CDF almost perfectly tracks the empirical CDF, outperforming both WED and WSD. Additionally, the polar CDF plot (Fig 13) confirms the ability of the WEED distribution to adapt to circular structures better than the classical models.

**Fig 14:**
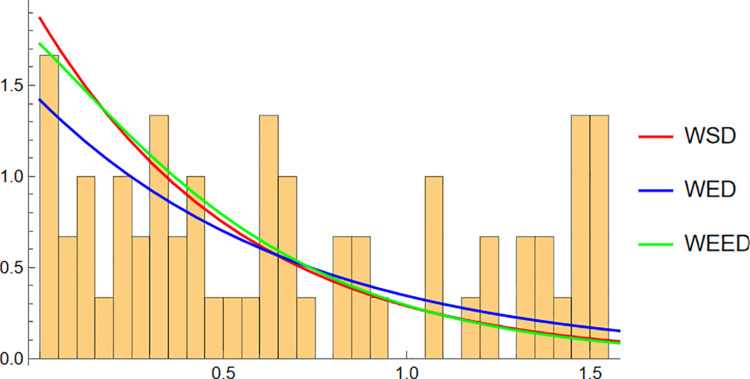
PDF of WEED, WED, and WSD compared with Fisher-B5 empirical histogram.

**Fig 15:**
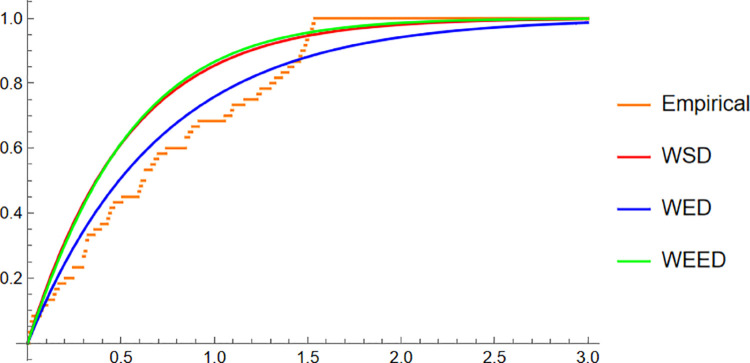
CDF of WEED, WED, and WSD compared with Fisher-B5 empirical CDF.

**Fig 16:**
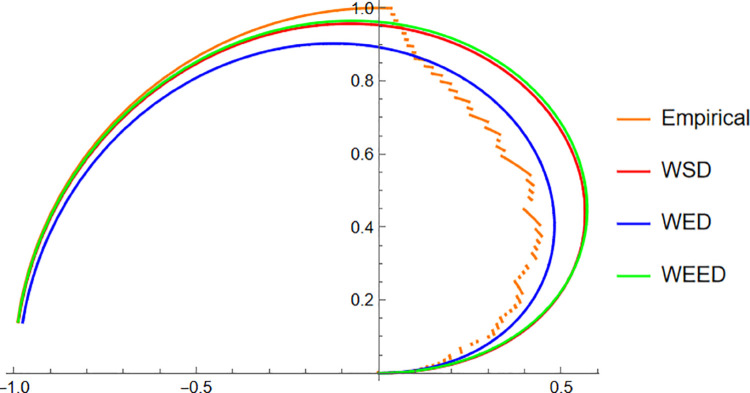
Polar CDF comparison of WEED, WED, and WSD for Fisher-B5 data.

Finally, for the Fisher5 data, the comparative analysis once again supports the superior performance of the WEED distribution. The PDF plot (Fig 14) shows a visibly improved fit, with the WEED curve following the empirical histogram more closely than its counterparts. The CDF plot (Fig 15) further confirms the enhanced cumulative fitting capability of the WEED model, while the polar CDF plot (Fig 16) illustrates that WEED provides a smoother and more accurate representation of the empirical circular cumulative pattern compared to WED and WSD.

Overall, the graphical comparisons across all three datasets consistently demonstrate that the WEED distribution offers significant improvements over the traditional wrapped distributions in terms of flexibility and goodness-of-fit. These results highlight the robustness and adaptability of the WEED model for modeling both linear and circular real-world data, thereby establishing its potential as a powerful alternative to existing wrapped distributions in practical applications.

## 14 Conclusions

In this paper, we introduced a novel circular distribution, the Wrapped Epanechnikov Exponential Distribution (WEED), which is constructed by wrapping the Epanechnikov exponential distribution onto the unit circle. The theoretical development included the derivation of the probability density function (PDF) and cumulative distribution function (CDF), as well as an in-depth exploration of the distribution’s characteristic function, trigonometric moments, reliability function, and hazard rate. Parameter estimation was carried out using the maximum likelihood estimation (MLE) method, and a comprehensive simulation study was conducted to evaluate the consistency of the MLEs. The mean and standard deviation of the distribution were also derived to provide further insights into its behavior.

To assess the practical performance of the WEED model, we applied it to three real-world circular datasets and conducted a detailed empirical comparison with two established models: the Wrapped Exponential Distribution (WED) and the Wrapped Shanker Distribution (WSD). The evaluation included visual comparisons using PDF, CDF, and polar CDF plots, in addition to statistical model selection criteria (AIC and BIC) and goodness-of-fit tests (Kolmogorov–Smirnov). Across all datasets, the WEED distribution consistently demonstrated superior fitting performance, both visually and statistically, confirming its robustness and flexibility in modeling circular data.

In conclusion, the WEED model offers a significant contribution to the field of directional statistics by providing a more adaptable and accurate alternative for modeling circular phenomena. Its theoretical richness, combined with its strong empirical performance, makes it a valuable tool for both theoretical research and practical applications. As a promising direction for future work, we suggest investigating a generalization of this model through the development of the Wrapped Quasi Epanechnikov Exponential Distribution, which may yield further insights and broader applicability.
